# Stimuli-Responsive Oligolysine-PEG Coatings for Reductive-Triggered Decomplexation

**DOI:** 10.1021/acspolymersau.5c00012

**Published:** 2025-05-22

**Authors:** Hugo J. Rodríguez-Franco, Artem Kononenko, Maartje M. C. Bastings

**Affiliations:** Programmable Biomaterials Laboratory, Institute of Materials, Interfaculty Bioengineering Institute, School of Engineering, Ecole Polytechnique Fédérale Lausanne, Lausanne 1015, Switzerland

**Keywords:** DNA origami nanoparticles, stabilizing coatings, stimuli-responsive polymers, reductive deprotection, biointerfaces

## Abstract

DNA origami nanoparticles (DONs) hold great potential for interacting with biological systems, yet their applicability is limited by nuclease activity and challenging ionic conditions in biological environments. Among various stabilization strategies, oligolysine-PEG coatings have emerged as a preferred option due to their straightforward implementation and protective capacity. However, their static nature restricts compatibility with dynamic DON systems and may hinder the functional availability of preincorporated bioactive cues. Here, we introduce a strategy to confer responsiveness to these coatings by incorporating labile disulfide bridges at defined positions within the oligolysine segments. Upon exposure to the characteristic reductive conditions of the cellular cytoplasm, these linkers undergo cleavage, weakening the multivalent electrostatic interactions between the coatings and DONs. Through the synthesis and characterization of distinct oligolysine-PEG variants with varying degrees of peptide segmentation, we confirm their ability to protect DONs under physiological conditions while enabling efficient decomplexation in reductive environments, observing differences in DON functional recovery depending on the number and positioning of the linkers. This work provides a foundation for developing responsive oligolysine-PEG coatings, broadening the functional scope and biomedical applicability of stabilized DONs.

## Graphical abstract

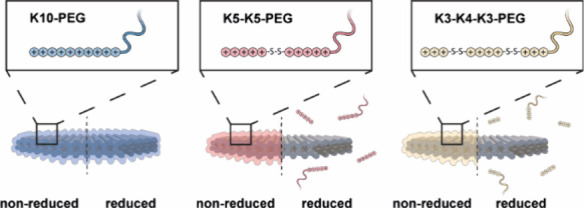

## Introduction

Structurally addressable DNA origami nanoparticles (DONs) hold great promise as tools to engage, interrogate, and manipulate biological systems due to their biocompatibility, ease and versatility of functionalization, and potential sensitivity to biological cues.^1,2^ DONs have already demonstrated utility across numerous functional domains, including catalysis, biomolecular detection and therapeutic delivery.^3–5^ Nonetheless, their overall applicability is significantly constrained by the harsh nuclease activity and challenging ionic conditions characteristic of biological environments, which drive the degradation and denaturation of DONs, underscoring the critical need for effective protective measures.^6–8^

Because of their straightforward implementation, coatings constitute the most widely embraced stabilization strategy, which has translated into the development of a prolific variety of alternatives, including polymeric, lipidic, proteic, and inorganic variants.^9–17^ While effective in providing protection, coatings result in extensive surface coverage that can potentially impair the intended performance of DONs. Although a few coatings have demonstrated the ability to retain site-specific functionalization poststabilization,^18,19^ they generally risk compromising the functional readiness of the preincorporated cues by limiting their accessibility. Moreover, the mainly static and nonadaptive nature of their complexation mechanisms makes them particularly incompatible with the dynamic requirements of many DON-based drug delivery systems, which often depend on stimulus-specific conformational switchability or location-specific biodegradability for effective cargo release.^20,21^

In line with the general trend in functional nanoparticle research,^22,23^ recent efforts in the field have explored stimuli-responsive coating designs to address some of these limitations, resulting in the emergence of two adaptive coatings that enable controlled disassembly upon light exposure. One design harnesses the cleavage of photolabile groups to disassemble BSA-dendron conjugates from their tethered spermine binding domains, restoring the binding properties of the DON-conjugated antibody fragments.^24^ The other system, based on cationic lipids, achieves complete coating decomplexation through the light-triggered loss of hydrophobic lipid tails, which are essential to the multivalent complexation driven by both electrostatic and hydrophobic forces. By implementing similar strategies, other stabilizing coatings leveraging multivalent interactions for complexation could potentially be engineered to exhibit stimuli-responsive behavior.^25^

Promising candidates for the integration of stimuli-triggered decomplexation strategies are the widely adopted oligolysine-poly­(ethylene glycol) (PEG) stabilizing coatings.^10^ Readily available and easy to implement, these coatings offer reliable protection of DONs in biological environments, which has led to their extensive characterization and effective application in a wide range of *in vitro* and *in vivo* settings.^26–32^ While unobtrusive in some scenarios,^30,32^ the nuclease protective role of the PEG chains can occasionally come at the cost of impairing DON functionality, with reports of entropic penalties affecting allosterically guided particle dimerization and reduced binding affinities to SPR or plate-immobilized receptors.^18,33^ In addition, these coatings rely on a 10-lysine block (K10) for efficient multivalent electrostatic complexation with DONs by spanning adjacent DNA helices, concurrently shielding the repulsive forces between them but adversely limiting the coating’s applicability to conformationally dynamic DON systems. Interestingly, the K10 segment was found to be optimal for the static complexation of these coatings, with both experimental and computational studies indicating significantly reduced binding affinities for shorter sequences and increased aggregation tendencies for longer ones, and it has remained the standard in follow-up work.^10,34^ This presents an opportunity to endow these coatings with controlled lability by introducing sensitive linkers within the oligolysine binding segments to modulate their length—and hence valency—in a stimulus-specific manner.

Stabilizing coatings for DONs intended for biomedical applications are better suited to respond to endogenous stimuli rather than exogenous ones. Since many therapeutic drugs exert their effects within the cytoplasm, the characteristic highly reducing environment of the cytosol provides a valuable internal cue for stimuli-responsive coatings, a mechanism widely leveraged by active cargo-delivery systems.^35,36^ This reducing environment is maintained by the abundant presence of glutathione (GSH), a tripeptide consisting of glutamate, cysteine, and glycine.^37^ As the most important antioxidant synthesized by cells, GSH is present in the cytosol at concentrations ranging from 1 to 10 mM, depending on the cell type and health status, in sharp contrast to extracellular levels that are 1000-fold lower.^38,39^ The design of reduction-sensitive nanosystems often involves incorporating simple disulfide linkers, which can be readily cleaved by the thiol group of the GSH cysteine residue.^40^ Labile disulfide bridges have already been successfully harnessed by cell-internalized DNA assemblies,^41–44^ and they have also been employed in PEG-polycation copolymers for DNA complexation and delivery, enabling release under reductive intracellular conditions via alternative mechanisms.^45,46^ In addition, their incorporation into specific cationic peptides has generally preserved bioactivity.^47^

Building on these concepts, here we explore the possibility of endowing oligolysine-PEG coatings with responsiveness to reductive conditions through the straightforward introduction of disulfide bridges at defined positions of the peptide segments, which we hypothesize will enable stimulus-specific coating decomplexation. By synthesizing oligolysine-5kPEG coatings with varying numbers of disulfide linkers, characterizing them, and assessing ligand functional recovery postdecomplexation, we aim to provide a modular platform that demonstrates the feasibility of rendering these coatings functionally labile. This work seeks to expand the applicability of oligolysine-PEG coatings, contributing to a more precise and temporally controlled performance of stabilized DONs, while supporting their integration into structurally switchable DON systems.

## Results and Discussion

We selected oligolysine­(K)-PEG coatings consisting of 10 lysine residues and 5kPEG chains as the central system of study due to their most widespread adoption and their higher propensity to interfere with DON functionality compared to shorter PEG variants, making them particularly well-positioned to benefit from enhanced responsiveness. Guided by the hypothesis that fragmenting the oligolysine block would weaken the multivalent electrostatic interactions with the DONs and favor decomplexation, we engineered coatings integrating labile disulfide linkers along the oligolysine segments to confer responsiveness to reductive conditions. To investigate the feasibility and functional implications of this modification, we synthesized and compared three distinct coating designs representing a systematic progression of valency disruption, each reflecting different stages of multivalent binding attenuation (Figure 1a). The first, serving as a negative control, consisted of a continuous 10-lysine sequence without disulfide linkers (K10-PEG). The second incorporated a single disulfide bridge at the midpoint of the oligolysine chain, resulting in two 5-lysine segments upon reduction (K5-PEG). The third introduced two disulfide linkers positioned symmetrically at equidistant lysine residues from both termini, yielding an intermediate 4-lysine fragment and two 3-lysine segments upon cleavage, one of which remained attached to the PEG block (K3-PEG).

**Figure 1:**
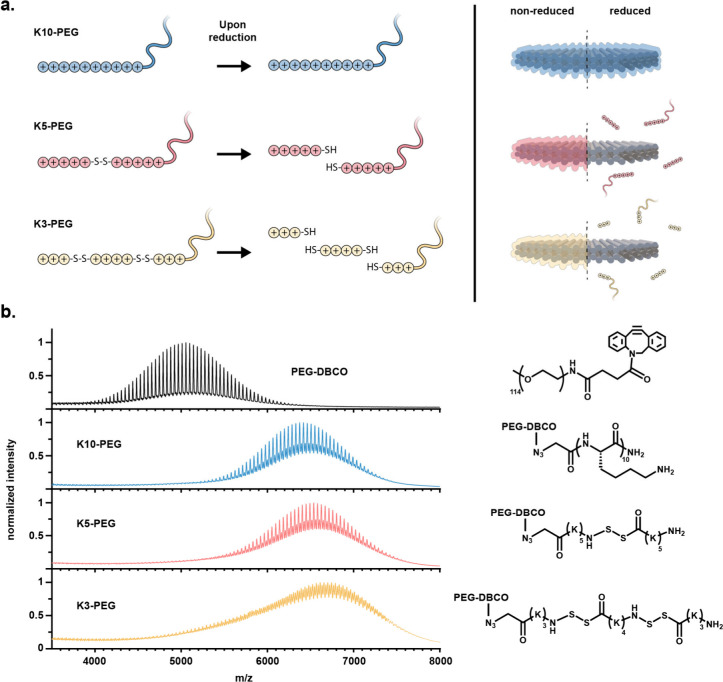
Design and molecular characterization of stimuli-responsive oligolysine-PEG coatings. (a) Schematic representation of the engineered oligolysine-PEG coatings, illustrating the site-specific incorporation of disulfide bridges, with an increasing degree of oligolysine segmentation across variants, to enable controlled fragmentation and decomplexation from DONs upon exposure to a reductive trigger. (b) MALDI-TOF mass spectra of the synthesized oligolysine-PEG coatings, with the PEG-DBCO precursor as a reference to highlight the spectral shifts (left), and their corresponding molecular structures (right).

We synthesized the different oligolysine blocks using manual solid-phase peptide synthesis (SPPS), a fast and accessible approach with potential for automation. To facilitate downstream conjugation, we selected the strain-promoted azide–alkyne cycloaddition (SPAAC) reaction due to its biorthogonal nature, compatibility with biological systems, and well-established use in bioconjugation strategies. As part of this design, we introduced azido-functionalization at the N-terminus of the oligolysine peptides through coupling with azidoacetic acid. The identity and high crude purity of the synthesized peptides were confirmed by matrix-assisted laser desorption ionization-time-of-flight (MALDI-TOF) mass spectrometry (Figure S1). We then conjugated the azido-functionalized oligolysine peptides to 5kPEG-dibenzocyclooctyne (DBCO) via SPAAC and purified the resulting conjugates using reverse-phase high-performance liquid chromatography (RP-HPLC) (Figure S2). MALDI-TOF spectra displayed mass shifts matching the expected increases upon conjugation of each peptide variant to PEG-DBCO, consistent with successful coupling (Figure 1b).

We then proceeded to evaluate the intrinsic interaction and stabilization of DONs. As a DON-based platform, we used a multilayered, disk-shaped particle (60 nm diameter, 7 nm thickness) purposely designed to interface with biological systems and extensively studied in complexation with various oligolysine-PEG coatings.^18,48–51^ Prior to use, the proper folding and purification of the variants, which differ in their incorporated functionalities, were verified via agarose gel electrophoresis (AGE), observing successful fluorophore incorporation and efficient removal of unbound strands (Figure S3). To assess the complexation efficiency of the synthesized coatings, we incubated the DONs at N/P ratios ranging from 0.25 to 1 (nitrogens in amines to phosphates in DNA) and performed AGE for both qualitative and quantitative assessments, benchmarking them against the commercially available, nonresponsive K10–5kPEG coating (cPEG) (Figure 2a). Of note, the inclusion of both cPEG and K10-PEG coatings as nonresponsive controls enables a direct comparison that isolates the impact of structural differences introduced during synthesis. All the synthesized coatings exhibited similar shifts in mobility compared to their cPEG counterparts, indicating successful coating complexation and charge compensation. However, in contrast to cPEG, the synthesized coatings showed progressively greater band smearing with increasing numbers of disulfide bridges, suggesting less efficient complexation. Gel densitometric quantification revealed that the control K10-PEG coating bound the DONs with about 20% lower efficiency compared to cPEG, while reductions of 30% and 40% were observed for the K5-PEG and K3-PEG coatings, respectively. Most probably, the cooperative electrostatic effect of 10-lysine chains is lost when present in shorter segments. Furthermore, the bulkier DBCO linker between peptide and PEG blocks could sterically compromise the complexation with the first amino acid and the DON surface.^52^ All coatings similarly preserved the overall morphology of the DONs, as confirmed through transmission electron microscopy (TEM) imaging (Figure 2b).

**Figure 2:**
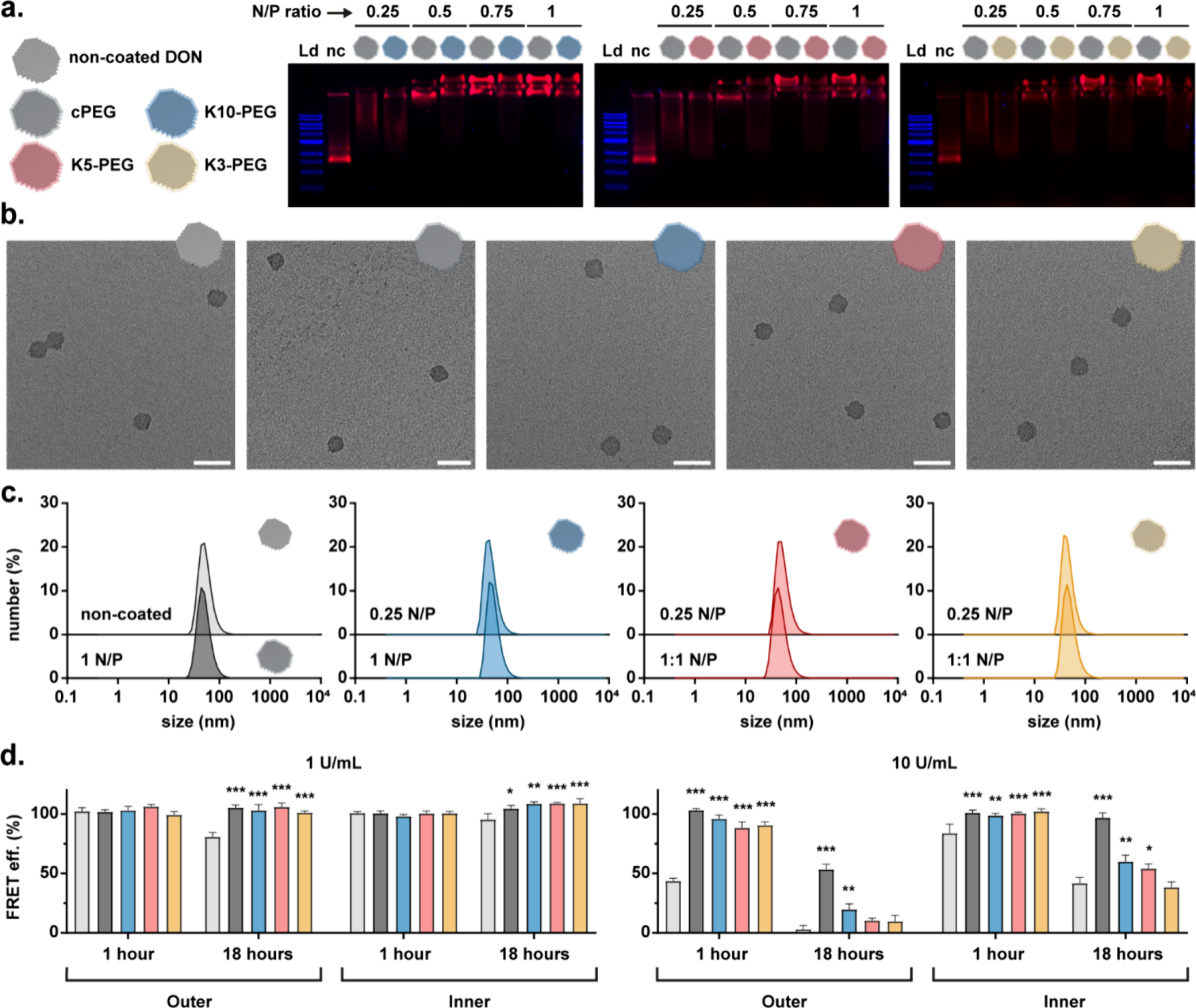
Intrinsic interactions and stabilizing effects of oligolysine-PEG coatings on DONs. (a) Assessment of coating complexation at different N/P ratios for the synthesized oligolysine-PEG coatings via AGE (1% gel, 90 min run). Ld: 1 kB ladder; nc: noncoated DON. Cy5 signal is shown in red, SybrSafe in blue. (b) TEM analysis of DON morphological preservation postcoating at a 1:1 N/P ratio for 5 nM DONs. Scale bars represent 200 nm. (c) Number-based size distribution of 5 nM DONs in FoB coated at 0.25 and 1 N/P ratios, measured by DLS. (d) FRET-based assessment of DON structural integrity (edges: Outer; core: Inner) at 37 °C for 5 nM DONs after exposure to 1 U/mL (left) and 10 U/mL (right) of DNase I for varying periods of time. The relative FRET efficiencies of coated DONs are shown in comparison to DMEM-incubated counterparts. Data are represented as mean ± SD, *n* = 3 in a single experiment. Statistical significance was assessed using ordinary one-way ANOVA followed by Dunnett’s multiple comparisons test, with comparisons made relative to the noncoated group. *p* ≤ 0.033; *p ≤ 0.002; *p* ≤ 0.001. Only statistically significant differences are annotated.

In view of the distinct complexation efficiencies observed, we evaluated their potential implications on the stabilization offered by each coating variant. The colloidal stability of the stabilized DONs in folding buffer (FoB) containing 18 mM of MgCl_2_ was analyzed at room temperature via dynamic light scattering (DLS) measurements across the distinct N/P ratios (Figure 2c and Figure S4). FoB was selected as the measurement buffer due to previously observed differences in variant behavior under these conditions.^50^ In all cases, the DONs remained monomeric, confirming that even the lowest N/P ratio was sufficient to prevent aggregation and corroborating that the observed shifts in mobility during AGE analysis were primarily due to charge compensation rather than potential aggregation-related effects, which could otherwise impact their biological behavior by interfering with structural integrity and/or hindering DON function.

Similarly, we examined the structural stabilization conferred by the coatings through nuclease-digestion kinetics assays, using a robust FRET reporter system previously optimized for this DON platform.^50^ Six Cy3-Cy5 FRET pairs were integrated across the particles, either at the center (Inner DON) or near the edges (Outer DON), to identify potential trends in digestion susceptibilities. For these assays, 5 nM DONs were coincubated with DNase I at 1 U/mL and 10 U/mL, and fluorescence readouts were collected at specific time points at 37 °C over a 24-h incubation (Figure 2d). The 1 U/mL concentration represents maximum physiological levels in serum,^7^ while the supraphysiological 10 U/mL condition was used to better distinguish variations between coatings. Additional assays were performed in 10% FBS-containing DMEM to mimic cell culture conditions (Figure S5).

Obtained results revealed a general tendency for noncoated DONs to degrade under all tested conditions, with varying susceptibility depending on the environment. In contrast, all coatings appeared to protect the structural integrity of the DONs under physiological conditions throughout the duration of the assay. However, under supraphysiological conditions, differences potentially related to the above-mentioned complexation strengths were visible: the cPEG coating provided a better protection compared to the synthesized counterparts, with K10-PEG stabilizing the DONs more effectively than the disulfide-containing versions. This trend closely aligns with the complexation efficiencies of the respective coatings, linking lower binding efficiencies to reduced nuclease restriction. Furthermore, while the structural stabilization offered by the synthesized coatings against high nuclease concentrations was evident at short time points, prolonged exposure resulted in minimal protective benefits, particularly for K3-PEG. This behavior suggests that the weakened electrostatic interactions are more prone to complete disruption by nuclease activity, which more readily exposes the underlying DNA to degradation. Additionally, the outer regions of the DONs exhibited greater susceptibility to digestion than the inner ones, a pattern consistent with previous oligolysine-PEG studies and thought to be primarily linked to the greater structural flexibility of the peripheral areas.^50^

Expanding on the characterization of their binding and stabilization properties, we next evaluated the responsiveness of the synthesized oligolysine-PEG coatings under reductive conditions to assess their potential for controlled decomplexation and functional recovery of DONs. Exposure to 10 mM GSH at 37 °C and physiological pH was used as a standardized reducing stimulus within biologically relevant environments, reflecting the maximum cytoplasmic concentration of this compound.

We verified the lability of the engineered coatings and monitored the kinetics of disulfide bond disruption via MALDI-TOF analysis immediately following reductive exposure (Figure 3a). The coatings were tested at amounts equivalent to those required to coat 5 nM DONs at a 1:1 N/P ratio. Complete disulfide bond cleavage in the labile coatings (K5- and K3-PEG) was achieved within 1 h—the earliest time point tested—with no detectable changes in the structural composition of the nonlabile K10-PEG control. This rapid cleavage was similarly observed under intermediate (5 mM) and lower (1 mM) GSH conditions (Figure S6), spanning the full range of cytoplasmic concentrations across distinct cell types, demonstrating the system’s potential to efficiently respond in these environments. Since a slower reductive process could better reveal the sensitivity differences between the K5- and K3-PEG coatings, we exposed them to reductive conditions at a more acidic pH 3, which further promotes the less reactive, protonated state of the cysteine thiol group in GSH (p*K*_a_ ∼ 8.6)^53^ (Figure S7). However, both coatings displayed similar disruption kinetics, reaching near-complete disulfide bond cleavage at 24 h under these conditions. Additionally, a 10-fold increase in coating concentration did not noticeably alter the reduction kinetics, suggesting comparable behavior across practical coating levels (Figure S7).

**Figure 3:**
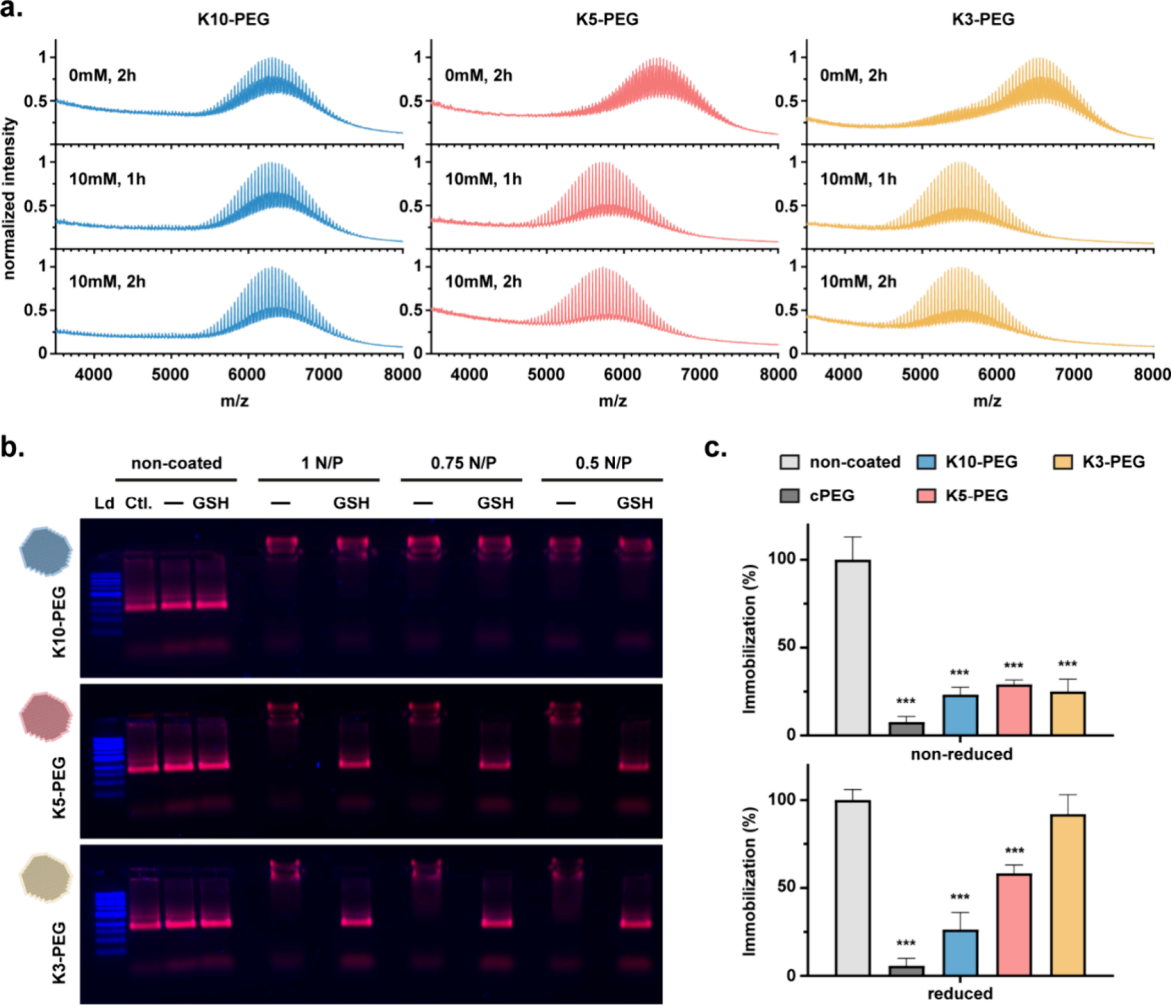
Functional lability and decomplexation of stimuli-responsive oligolysine-PEG coatings. (a) MALDI-TOF mass spectra of distinct oligolysine-PEG coatings exposed to 10 mM GSH at 37 °C for up to 2 h, revealing lability through changes in mass distribution. (b) Evaluation of coating decomplexation via AGE (1% gel, 90 min run) after exposure of 5 nM DONs, previously coated at different N/P ratios, to either none (—) or 10 mM GSH at 37 °C for 1 h. Ld: 1 kB ladder; Ctl: noncoated DON in FoB. Cy5 signal is shown in red, SybrSafe in blue. (c) Postdecomplexation functional recovery assessed via an immobilization assay on a streptavidin-coated plate using biotinylated DONs (5 nM, 1:1 N/P coating) after incubation under nonreductive conditions (top) or with 10 mM GSH (bottom) at 37 °C for 1 h. Immobilization (%) was determined via fluorescence readout and expressed as % relative to noncoated DONs. Data are represented as mean ± SD, *n* = 3 in a single experiment. Statistical significance was assessed using ordinary one-way ANOVA followed by Dunnett’s multiple comparisons test, with comparisons made relative to the noncoated group. *p* ≤ 0.033; *p ≤ 0.002; *p* ≤ 0.001. Only statistically significant differences are annotated.

To assess the functional responsiveness of the coatings and their associated decomplexation behavior from DONs, we performed qualitative AGE shift mobility assays on DONs coated at distinct N/P ratios following 1 h exposure to GSH or β-mercaptoethanol, a more nucleophilic reducing agent serving as a positive control due to its aggressive reduction kinetics (Figure 3b and Figure S8). The nonlabile K10-PEG control remained stably complexed under all tested conditions, as evidenced by its retention in the well due to the sustained charge compensation from the coating. In contrast, the K5-PEG and K3-PEG coatings exhibited clear decomplexation across all tested N/P ratios, with the DONs regaining a mobility comparable to that of the noncoated particles. Interestingly, the decomplexation triggered by the reducing agents was more efficient than that achieved through the standard use of the competitive chondroitin sulfate (ChS) polyanion, as indicated by the greater mobility recovery (Figure S9). Furthermore, ChS added after reductive exposure did not induce further changes in DON mobility, suggesting apparent complete decomplexation of the labile coatings under the conditions tested.

Finally, to further validate the functional implications of the reductive decomplexation, we evaluated a scenario where oligolysine-PEG coatings were reported to hinder key functionalities of DONs. Specifically, we examined the ability of biotin-functionalized DONs to immobilize on streptavidin-coated plates. For this purpose, 7 biotin molecules were integrated into the surface plane of Cy5-functionalized DONs, and the resulting structures were stabilized at a 1:1 N/P ratio using the distinct oligolysine-PEG coatings. Immobilization of noncoated and coated DONs following a 1-h exposure to reductive conditions was then measured via fluorescence readout (Figure 3c). The results from the nonreduced control conditions confirmed the practical functional inhibition of the biotin moieties for cPEG-coated DONs. Similarly, without reduction, all synthesized coatings hindered immobilization while retaining ∼ 25% of the original binding capacity. Upon pre-exposure of the stabilized DONs to reducing agents, the nonlabile coatings maintained similar binding levels, while the labile K5- and K3-PEG coatings exhibited a notable increase in immobilization efficiency. Specifically, the K3-PEG variant facilitated near-complete functional recovery, whereas K5-PEG responsiveness restored DON binding to streptavidin-coated plates to approximately 60% of their original capacity.

The slightly lower inhibition of DON binding provided by the synthesized coatings compared to cPEG under nonreducing conditions aligns with their relatively lower nuclease restriction and can also be linked to their overall lower binding affinities. In addition, the performance differences observed between the labile coatings in this assay highlight the influence of the number and positioning of the disulfide bridges, with the K3-PEG coating, which splits the oligolysine block into shorter segments upon cleavage, enabling greater functional recovery. These previously unrecognized variations further suggest that coating release during the prior decomplexation assessment was partially influenced by AGE-specific electrophoretic or hydrodynamic forces, which may have contributed to the complete disruption of weakened electrostatic interactions, thereby obscuring distinctions between the labile coatings.

## Conclusion

Through a straightforward and accessible engineering step, we present an adaptable oligolysine-PEG platform that enables time- or location-specific responses, supporting the functional stabilization of dynamic systems previously incompatible with conventional coatings (e.g., conformationally switchable or degradation-based drug delivery DON constructs). Our findings indicate that the disruption of multivalent electrostatic interactions upon disulfide cleavage, while ultimately depending on the number and positioning of the labile linkers, is sufficient to drive the controlled decomplexation of the synthesized coatings and enable stimulus-specific functionality in scenarios where they previously hindered DON performance. In particular, our results demonstrated effective DON stabilization under physiological conditions and confirmed ligand functional recovery following reductive input, with the K3-PEG coating achieving superior performance due to its higher degree of segmental fragmentation.

While the disulfide bridge strategy is particularly suited to the reducing cytoplasmic environment, DON cellular internalization typically occurs through endosomal pathways. Interestingly, endocytic organelles also exhibit minor reducing activity, primarily through enzymes such as TRX-1, PDI-3, and GILT, rather than small molecules like GSH.^41,54^ Investigating whether the coatings sterically hinder enzymatic reduction or maintain responsiveness within such compartments could help expand their applicability to diverse cellular settings, including targeted endosomal and lysosomal processes. Notably, cytosolic delivery could enable immunostimulatory applications such as cGAS-STING activation,^55^ where coating removal may be required to restore DNA accessibility. Furthermore, other biologically relevant environments characterized by altered redox states, such as hypoxic bacterial infection sites, could also be leveraged, as bacteria often accumulate high levels of GSH through glycolysis-driven metabolism.^56^

Our system is a modular platform capable of integrating other stimuli-responsive linkers, such as pH-, enzyme- or reactive oxygen species (ROS)-labile ones, provided they are neutral enough to allow effective complexation. For example, linkers responsive to ROS may be particularly relevant for tumor-targeted applications, as these environments are characterized by elevated ROS levels.^57^ Given that DONs passively accumulate in tumors via the enhanced permeability and retention (EPR) effect,^58^ such coatings could enable controlled uncoating at the disease site. We offer a foundation for responsive oligolysine-PEG strategies and seek to inspire further designs aimed at developing functional coatings that enhance the performance and broaden the applicability of stabilized DONs across diverse biomedical settings.

## Materials and Methods

### Solid Phase Peptide Synthesis (SPPS) of Labile Oligolysines

Labile oligolysines were synthesized via Fmoc-based SPPS on Rink amide MBHA resin (Sigma-Aldrich, loading 0.54 mmol/g). The synthesis was carried out manually in 10 mL reaction columns (Torviq) fitted with frits under gentle agitation. 100 mg of resin was first swollen in DMF (abcr GmbH) for 1 h, followed by Fmoc deprotection using 20% piperidine (Thermo Scientific) in DMF (v/v) for 7 min (2×). After thorough washing with DMF, DCM (Sigma-Aldrich), and NMP (abcr GmbH) (3× each), amino acid coupling was performed in two rounds per residue, with 5 equiv of of Fmoc-Lys­(Boc)-OH (Sigma-Aldrich), 4.5 equiv of of HBTU (Abcr GmbH), and a base mixture of 1.25 equiv of DIPEA (abcr GmbH) and 1.95 eq 2,6-lutidine (Thermo Scientific) in NMP. Each round was incubated for 30 min at room temperature, followed by washing with DMF (3×). Each peptide incorporated a total of ten lysine residues through this iterative coupling process. To cap unreacted amino groups, 5% acetic anhydride (Sigma-Aldrich) and 6% 2,6-lutidine in DMF were applied after each coupling cycle, followed by a 7 min incubation at room temperature. The resin was then thoroughly washed with DMF, DCM, and DMF again (3× each) to remove excess reagents before the next deprotection step. Coupling efficiency was monitored via the ninhydrin test. For labile oligolysine constructs, disulfide linkers were incorporated by coupling 5 equiv of of Fmoc-NH-ethyl-SS-propionic acid (MedChemExpress) in a single 2-h coupling round at room temperature. For final azido-functionalization at the N-terminus, 5 equiv of of azidoacetic acid (TCI) was coupled in NMP over two successive rounds of 1-h incubations at room temperature. After synthesis, the resin was thoroughly washed (3 × DMF, 5 × DCM) and dried under vacuum. Peptides were cleaved from the resin in TFA (Sigma-Aldrich) for 2 h at room temperature. The crude peptides were precipitated in cold diethyl ether (Honeywell), collected by centrifugation, and dried under vacuum. Crude peptides were then resuspended in water and lyophilized to remove residual solvents. Matrix assisted laser desorption ionization-time-of-flight mass spectrometry (MALDI-TOF MS) confirmed peptide identity and high crude purity, eliminating the need for further purification.

### PEG-Peptide Conjugation and Purification

The synthesized oligolysine peptides were conjugated to mPEG-DBCO (5 kDa) (TargetMol) via strain-promoted azide–alkyne cycloaddition (SPAAC). 5kPEG-DBCO, initially resuspended in water to a concentration of 10 mM, was mixed with a 2-fold molar excess of oligolysine peptides. The peptides were resuspended in water to a concentration of 30 mM. Each reaction mixture was prepared in a total volume of 240 μL and included 20 mM HEPES buffer (Thermo Scientific). The reaction proceeded overnight at room temperature. The resulting compounds were purified by reverse-phase high-performance liquid chromatography (RP-HPLC) on a ThermoFisher UltiMate 3000 chromatograph using a Hypersil GOLD C18 column. The following gradient was applied for the purification of peptide-PEG conjugates: 0–5% acetonitrile (Carlo Erba) in 0.1% aqueous TFA over 2 min, followed by 5–90% acetonitrile in 0.1% aqueous TFA over 24 min. The fractions of interest were collected and lyophilized. MALDI-TOF mass spectrometry confirmed the identity of the purified conjugates.

### Synthesis and Purification of DONs

The p7560 DNA scaffold was sourced from Tilibit, and sequence-specific staple strands were custom-ordered from Integrated DNA Technologies. DONs were prepared for assembly by mixing 10 nM of the p7560 scaffold with a 10-fold molar excess of nonmodified staple strands, a 5-fold molar excess of fluorophore-functionalized strands^50^ and a 7-fold molar excess of biotinylated strands,^51^when required. The assembly was carried out in folding buffer (FoB) containing 5 mM Tris (Merck), 1 mM EDTA (PanReac AppliChem), 5 mM NaCl (Sigma-Aldrich), and 18 mM MgCl_2_ (Sigma-Aldrich), adjusted to pH 8, in a total reaction volume of 50 μL. The folding process involved thermal annealing using a Biometra TRIO Analytik Jena thermocycler, where the reaction mixture was initially heated to 80 °C for 5 min, then gradually cooled from 60 to 20 °C at a rate of −1 °C per hour. Postassembly, DONs were purified and concentrated through PEG precipitation. Annealed samples were mixed in a 1:1 (v/v) ratio with 2× PEG precipitation buffer (15% PEG8000, 0.5 M NaCl, 5 mM Tris, 1 mM EDTA, and 18 mM MgCl_2_) and incubated for 20 min at room temperature. The solutions were then centrifuged at 16,000 rcf for 30 min at 20 °C, and the resulting supernatant was discarded. The DON-containing pellet was resuspended immediately in FoB to achieve a final concentration of 30 nM, as determined by measuring UV absorbance at 260 nm using a NanoDrop spectrophotometer (Quawell Q9000). Samples were stored at 4 °C until further use.

### Kx-PEG Coating of DONs

Stock solutions of DONs were mixed with the respective oligolysine-5kPEG coating solutions (benchmark material obtained from Alamanda Polymers) in a 1:1 (v/v) ratio. 1 mM coating stocks, initially resuspended in water, were first diluted in folding buffer (FoB) to achieve the desired working concentrations for a target N/P ratio of 1:1 (nitrogens in amines to phosphates in DNA), unless stated otherwise. The mixtures were then incubated at room temperature for 1 h to ensure effective coating. Noncoated DONs were diluted under identical conditions for comparative analysis.

### Analytical AGE

The folding and purification quality of DONs, along with coating complexation and decomplexation, were visually evaluated using AGE. A 1 kB DNA ladder (New England BioLabs) served as a reference marker. Unless otherwise specified, an equivalent amount to 5 μL of 10 nM samples was mixed with 6× MassRuler loading dye (Thermo Scientific) before being loaded onto a 1% (w/v) agarose gel. The gel was prepared with SYBR Safe DNA stain (1×, Invitrogen), TBE buffer (0.5×, Thermo Scientific), and 8 mM MgCl_2_ (Sigma-Aldrich). Electrophoresis was conducted at 70 V for 90 min in an ice bath, and gel images were captured using a Bio-Rad ChemiDoc MP imaging system.

### Assessment of DON Colloidal Stability by DLS

Non-Cy5-functionalized DONs, initially at a concentration of 30 nM, were stabilized with their respective coatings at N/P ratios of 0.25, 0.5, 0.75, and 1:1. The samples were then diluted in folding buffer (FoB) to a final concentration of 5 nM in a total volume of 70 μL. After incubating for 10 min at room temperature, the entire sample was transferred into a disposable micro UV-cuvette (BRAND) and analyzed using a Zetasizer Nano ZS (Malvern Panalytical) equipped with a 633 nm He–Ne laser. DLS measurements were conducted at 25 °C in a backscatter configuration with a scattering angle of 173°. The resulting curves represent the average of three technical replicates, each recorded with an automatically determined number and duration of runs.

### FRET-Assisted Quantification of DON Integrity under Nuclease Activity

The protective performance of the different coatings was assessed by monitoring the digestion kinetics of stabilized DONs when exposed to either standard cell culture conditions or DNase I-containing DMEM solutions. The experiments included five DON designs, as previously introduced,^50^ consisting of two FRET-active structures differing in fluorophore placement (Inner or Outer) and three control designs that enable accurate quantification. Each DON design was treated equally and subjected to all tested conditions. DON stocks, initially concentrated at 30 nM, were stabilized with their respective coatings and diluted to a final concentration of 5 nM in a total volume of 30 μL. DMEM without phenol red (Gibco) was used as a negative control. For nuclease-active conditions, DMEM was supplemented with either 10% FBS (PAN-Biotech) or DNase I at final concentrations of 1 U/mL or 10 U/mL. 10× DNase I working solutions were prepared by diluting a 1 U/μL stock solution (Thermo Scientific) in DMEM to achieve the target enzyme concentrations. The prepared DON mixtures were plated in 384-well black plates (Greiner Bio-One) and incubated for 24 h in a Cytation 5 imaging reader (BioTek) to allow for nuclease digestion. Measurements were performed at 37 °C at predefined time points. The filter sets used, as well as the calculation of apparent FRET signals, were performed as described in previous studies.^50^ Relative FRET efficiencies were eventually calculated by normalizing against the corresponding nuclease-negative control conditions. Data were tested for normality using the Shapiro–Wilk test prior to analysis. Statistical significance was assessed using one-way ANOVA followed by Dunnett’s multiple comparisons test, with comparisons made relative to the noncoated group.

### TEM-Based Visualization of Coated DONs

The morphological impact of the coatings on DONs was visually assessed via TEM imaging. CF400-Cu grids (Electron Microscopy Sciences) were pretreated with a glow discharge process for 30 s at 1 mA to enhance sample adherence. A total of 8 μL of 5 nM DON solution was then deposited onto the grids and allowed to adsorb for 60 s. Excess liquid was carefully removed using filter paper. Samples were then stained with 1.5 μL of a 2% (w/v) uranyl acetate aqueous solution, followed by immediate blotting to eliminate excess stain. The grids were subsequently air-dried before imaging. TEM analysis was conducted using a Talos L120C instrument operating at 80 kV.

### Stimuli-Triggered Coating Decomplexation from DONs

For each sample, DON stock solutions at 30 nM were coated at the designated N/P ratios in a total volume of 5 μL with the respective coating solutions. The mixtures were then combined with 10 μL of PBS (Gibco) containing 6 mM MgCl_2_—added to maintain the structural integrity of uncoated DONs—supplemented with reduced l-glutathione (GSH, Sigma-Aldrich) to achieve a final reducing condition of 10 mM GSH. Prior to use, 1.5× GSH working solutions were adjusted to pH 7.4. GSH-negative control samples were diluted under identical conditions in either FoB or MgCl_2_-containing PBS. As a positive control, a final concentration of 5% β-mercaptoethanol (Thermo Fisher) was prepared following the same dilution approach. To promote decomplexation, samples were incubated at 37 °C for 1 h and subsequently analyzed via AGE. To prevent cross-contamination of reducing agents, the full sample volume was loaded into alternating wells.

### MALDI-TOF Mass Spectrometry Analysis

MALDI-TOF MS was used to confirm the identity of synthesized products and assess the lability of oligolysine-PEG coatings under reducing conditions. Lability tests were conducted using coating amounts and solvent compositions consistent with the decomplexation assays, under 10 mM, 5 mM, and 1 mM GSH or 5% β-mercaptoethanol. 1.5× GSH working solutions were adjusted to pH 7.4 unless otherwise specified. Samples were exposed to the reducing agents at 37 °C for defined periods and immediately analyzed. Sample preparation for MALDI-TOF MS involved mixing the solution 1:1 (v/v) with a 2,5-dihydroxybenzoic acid (DHB) matrix (15 mg/mL ethanol). Spectra were acquired using a MALDI-TOF/TOF AutoFlex Speed (Bruker) in positive ion mode with the direct TOF method.

### DON-Immobilization Assay

The functional recovery of DONs was evaluated by assessing the immobilization efficiency of distinctly treated, core-integrated Cy5-functionalized DONs in high-binding 96-well, half a rea plates (Greiner Bio-One). To enable DON binding, plates were precoated the day prior with 35 μL of a 300 nM streptavidin (Thermo Scientific) solution in PBS, spun down at 400 rcf for 30 s, and incubated overnight at 4 °C. On the day of the assay, DON stock solutions at 30 nM were coated with their respective coatings at a 1:1 N/P ratio, then diluted in PBS containing 18 mM MgCl_2_ to ensure the structural preservation of uncoated DONs. PBS solutions were either supplemented with GSH (final concentrations: 5 nM DON and 10 mM GSH) or left untreated as negative controls. Before use, 1.5× mM GSH working solutions were adjusted to pH 7.4. Samples were incubated at 37 °C for 1 h to promote decomplexation. Streptavidin-coated wells were washed twice with 75 μL of PBS and immediately blocked with 50 μL of 3% (w/v) BSA in PBS via a 30 min incubation at 37 °C to reduce nonspecific binding. Afterward, wells were washed twice with a solution matching the one used for DON dilution (excluding reducing agents), and the full volume of DON samples was transferred to the wells. Following a 1-h dynamic incubation at room temperature on a shaker to allow for DON binding, solutions were removed, and wells were washed twice before adding 100 μL of the same buffer for Cy5 fluorescence measurement. Fluorescence was recorded using a Cytation 5 imaging reader (BioTek) with a gain of 150 following a 5 min incubation at 90 °C. Data were tested for normality using the Shapiro–Wilk test prior to analysis. Statistical significance was assessed using one-way ANOVA followed by Dunnett’s multiple comparisons test, with comparisons made relative to the noncoated group.

## Supplementary Material



## Notes

MALDI-TOF of synthesized peptides, HPLC chromatograms, AGE of folded DONs, DLS measurements, FRET-based nuclease resistance assays, MALDI-TOF of reduced coatings, and AGE of coating decomplexation (PDF)
