# Supplementary material for: Stimuli-Responsive Oligolysine-PEG Coatings for Reductive-Triggered Decomplexation
Source: ACS Polym Au. 2025 May 22;5(4):343–52. doi: 10.1021/acspolymersau.5c00012 (PMC12355613; doi:10.1021/acspolymersau.5c00012)
Supplement: Supplementary file 1 [file lg5c00012_si_001.pdf]

**SUPPORTING INFORMATION**

**For**

# *Stimuli-Responsive Oligolysine-PEG Coatings for Reductive-Triggered Decomplexation*

*Hugo J. Rodríguez-Franco,[1] Artem Kononenko,[1] Maartje M.C. Bastings[1*].*

1 Programmable Biomaterials Laboratory, Institute of Materials, Interfaculty Bioengineering Institute, School of Engineering, Ecole Polytechnique Fédérale Lausanne, Lausanne, 1015, Switzerland

* Corresponding author: maartje.bastings@epfl.ch

## SUPPORTING FIGURES

**Figure S1|** Molecular structure verification of synthesized oligolysine peptides ........ 1

**Figure S2|** Purification of synthesized oligolysine-PEG coatings via HPLC ........ 2

**Figure S3|** Folding and purification of DONs analyzed by AGE ........ 3

**Figure S4|** Colloidal stability of coated DONs ........ 3

**Figure S5|** Structural stability of coated DONs under different nuclease conditions ........ 4

**Figure S6|** Lability of coatings under reductive conditions at physiological pH 7.4 ........ 5

**Figure S7|** Lability of coatings under reductive conditions at pH 3.0 ........ 6

**Figure S8|** Coating decomplexation AGE assay after exposure to β-mercaptoethanol ........ 7

**Figure S9|** Coating decomplexation AGE assay after exposure to chondroitin sulfate polyanion ........ 8

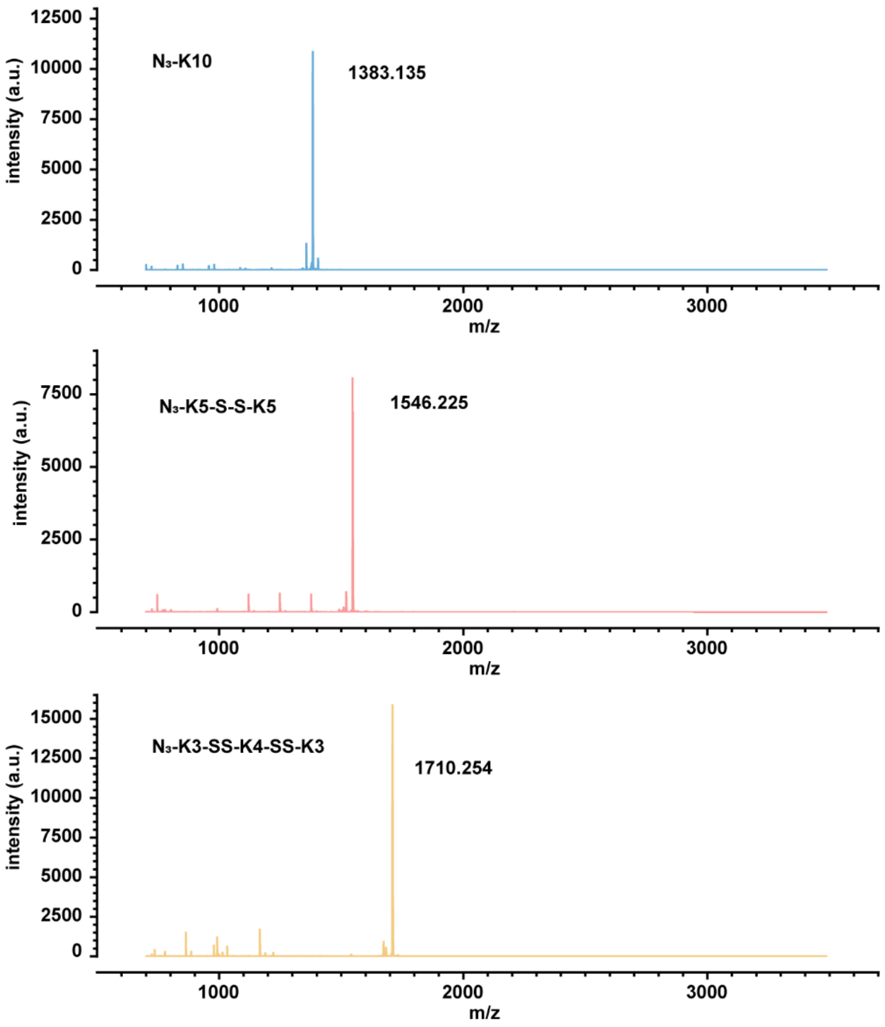


**Figure S1| Molecular structure verification of synthesized oligolysine peptides.**
MALDI-TOF mass spectra of the synthesized oligolysine blocks. The observed mass peaks correspond to the expected molecular weights of the respective oligolysine variants.

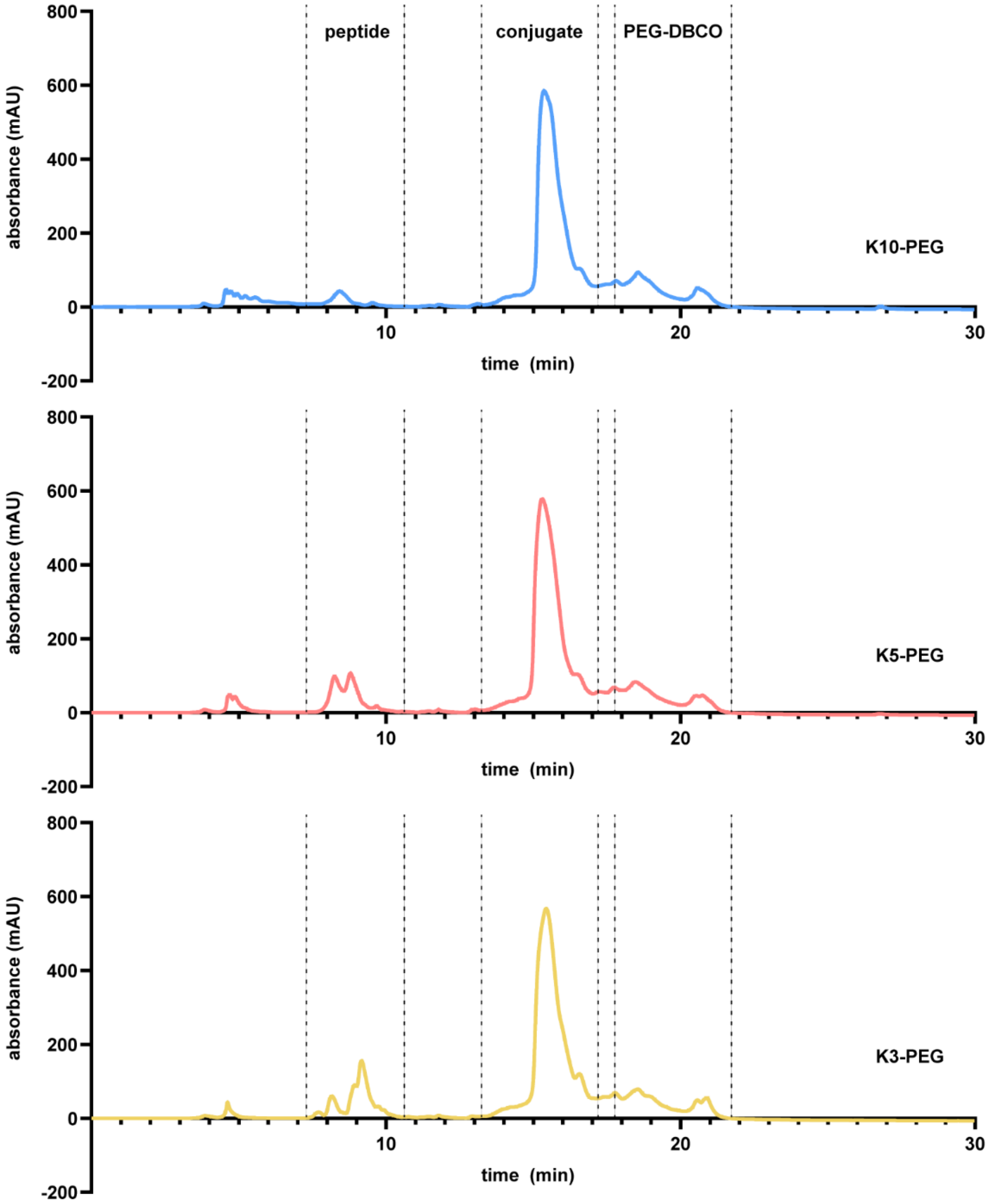


**Figure S2| Purification of synthesized oligolysine-PEG coatings via HPLC.**
HPLC chromatograms of oligolysine-PEG coatings during purification, highlighting the elution fractions corresponding to the final conjugates, as well as their peptide and PEG-DBCO precursors.

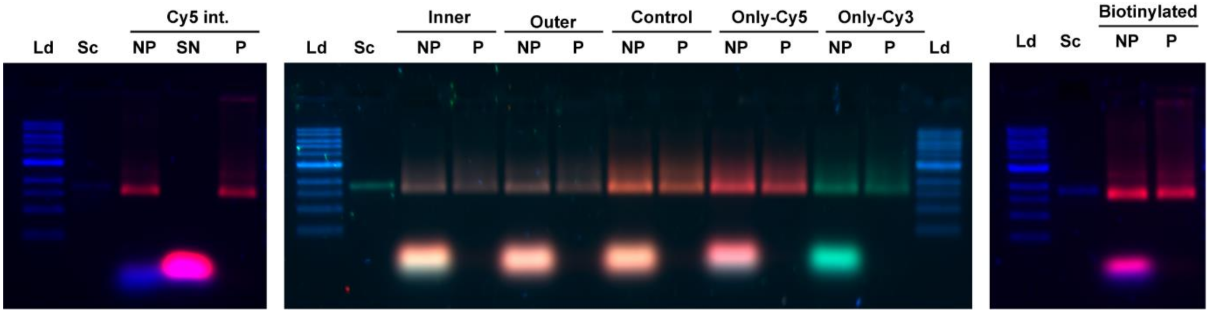


**Figure S3| Folding and purification of DONs analyzed by AGE.**
1% agarose gel (90 min run) verifying the proper folding and purified state of DONs, including the Cy5-integrated variant (left), FRET designs with a control where both FRET fluorophores are separated to prevent energy transfer (middle), and biotinylated disks (right). Cy5 signal is shown in red, Cy3 in green, and SybrSafe in blue. Ld: 1 kB ladder; sc: p7560 scaffold control.

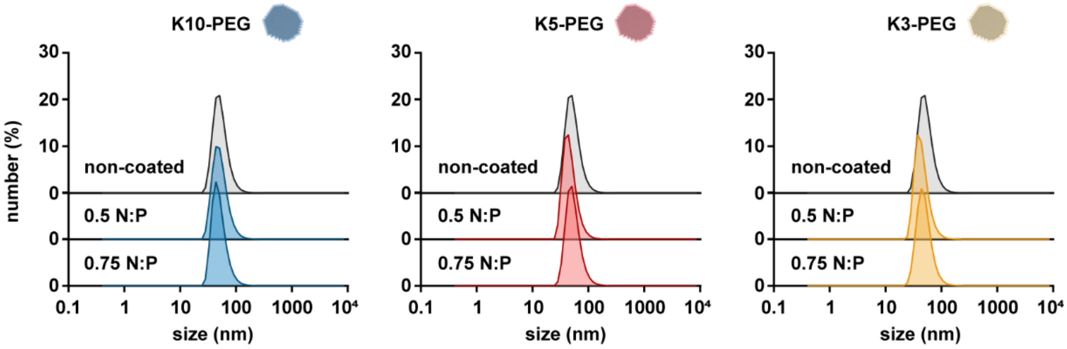


**Figure S4| Colloidal stability of coated DONs.**
Number-based size distribution of 5 nM DONs in FoB, coated at intermediate N/P ratios of 0.5 and 0.75 with the distinct variants, measured by DLS.

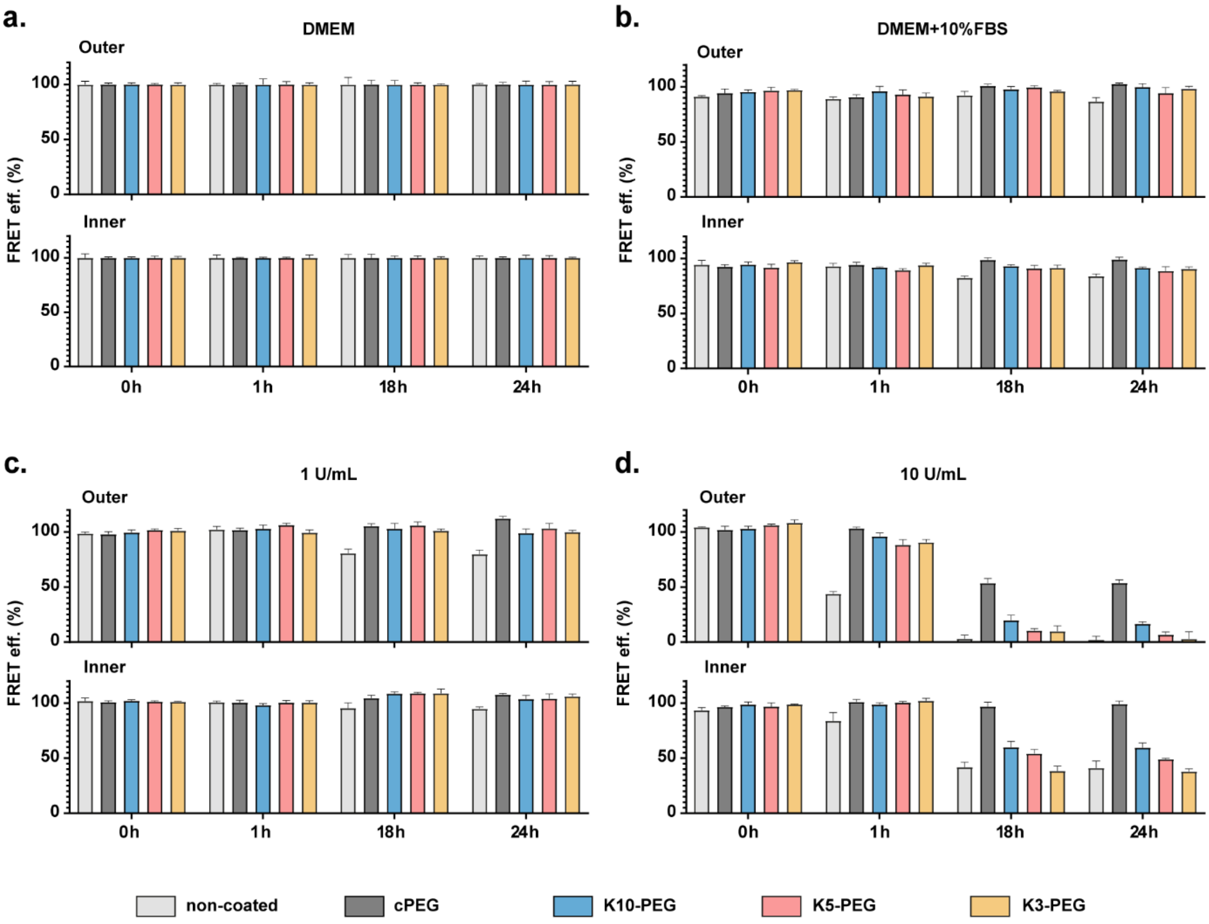


**Figure S5| Structural stability of coated DONs under different nuclease conditions.** FRET-based assessment of DON structural integrity (edges: Outer; core: Inner) at 37°C for 5 nM DONs after exposure to: **a,** DMEM without nuclease, used as a reference for comparison; **b,** DMEM supplemented with 10% fetal bovine serum (FBS) to mimic cell culture conditions; **c,** 1 U/mL DNase I; and **d,** 10 U/mL DNase I for varying periods of time. Data are presented as mean ± SD, n = 3 in a single experiment.

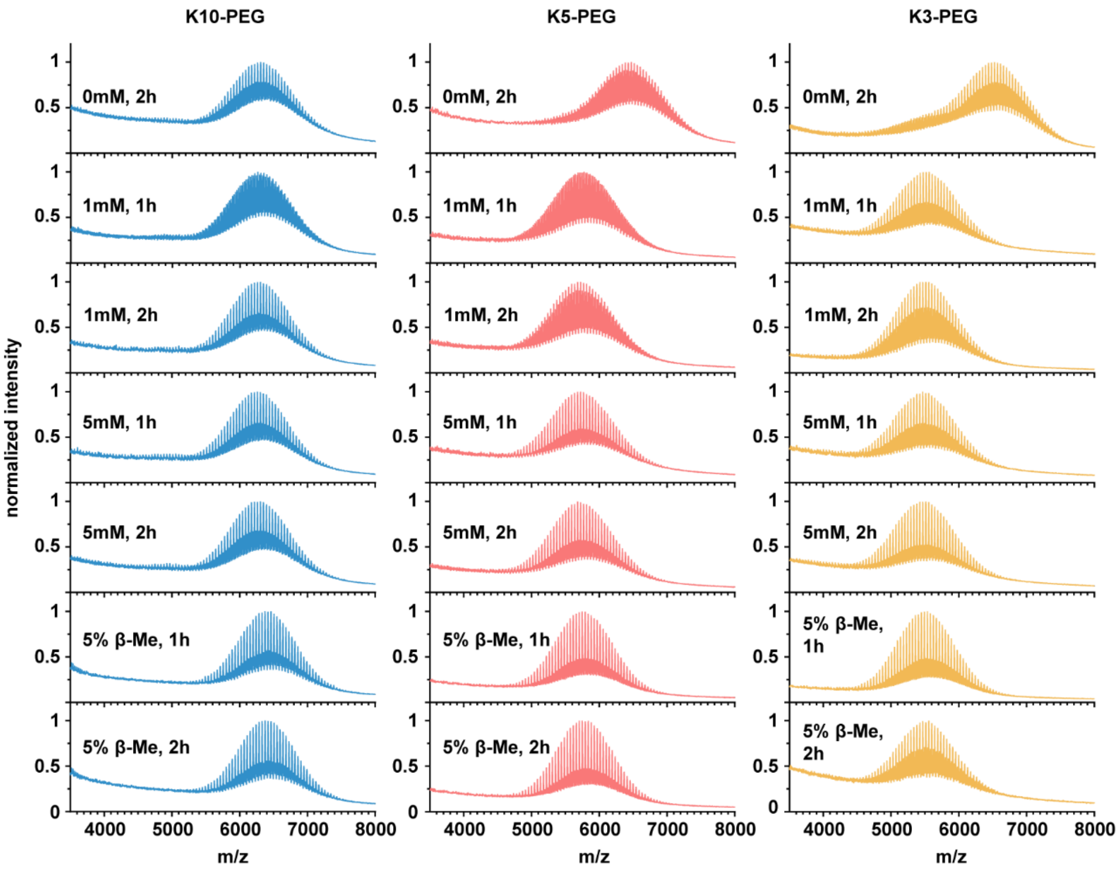


**Figure S6| Lability of coatings under reductive conditions at physiological pH 7.4.** MALDI-TOF mass spectra of the oligolysine-PEG variants incubated with 1 mM and 5 mM GSH, as well as 5% β-mercaptoethanol as a positive control, at 37 °C and pH 7.4 for up to 2 hours. Lability is revealed through changes in mass distribution.

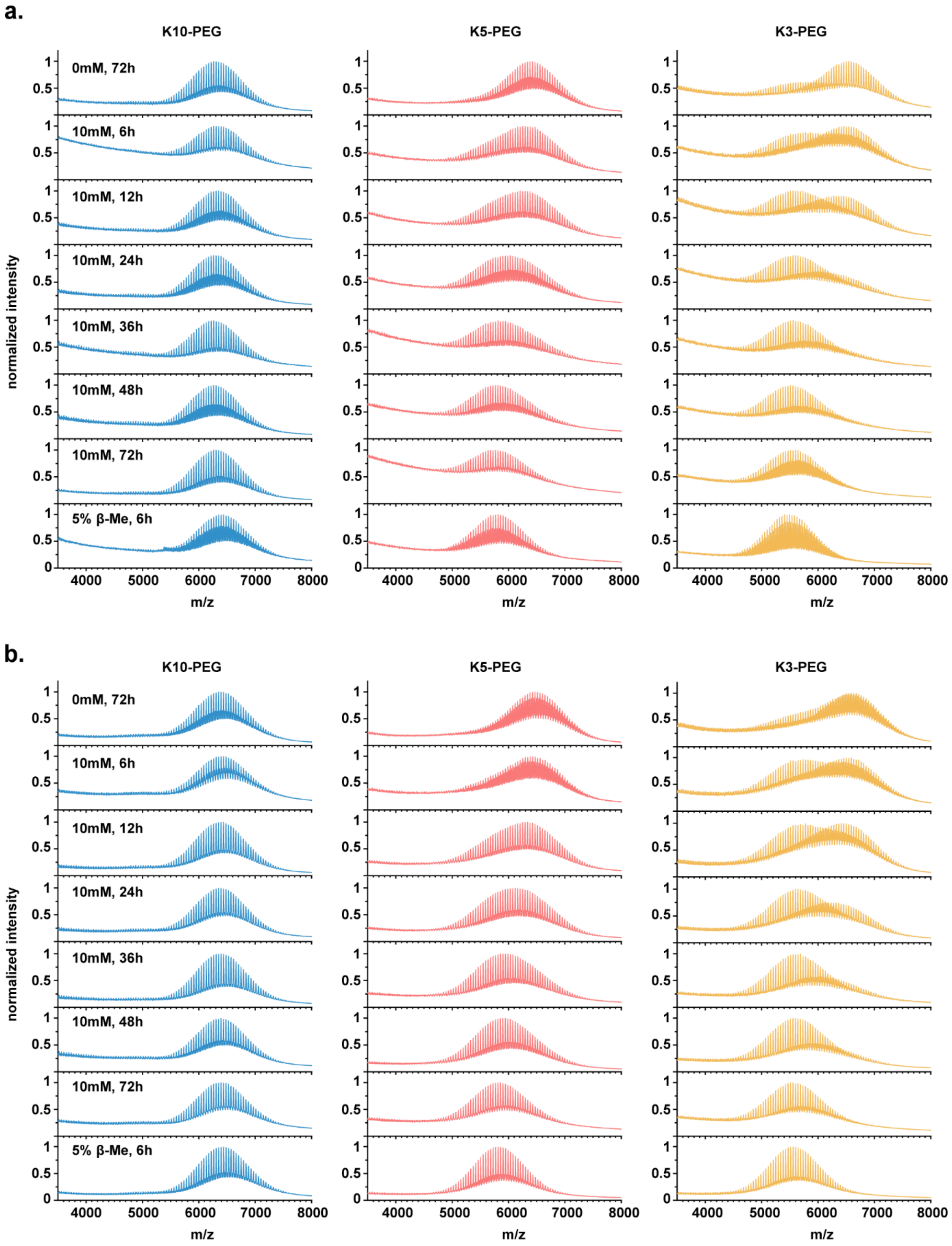


**Figure S7| Lability of coatings under reductive conditions at pH 3.0.**
MALDI-TOF mass spectra of the oligolysine-PEG variants exposed to 10 mM GSH at pH 3.0 to assess slower reaction kinetics. Measurements were performed at 1-fold (top) and 10-fold (bottom) coating concentrations relative to that required for a 1:1 N/P ratio, with incubation at 37 $^{o}$C lasting up to 72 hours. Lability is revealed through changes in mass distribution.

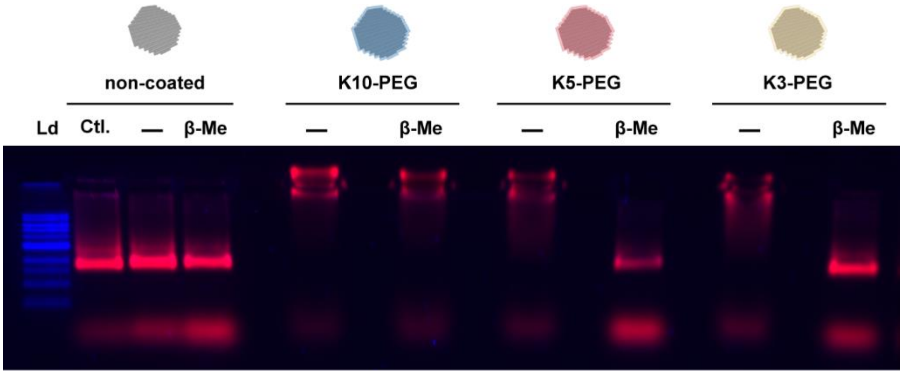


**Figure S8| Coating decomplexation AGE assay after exposure to β-mercaptoethanol.**
1% agarose gel (90 min run) evaluating coating decomplexation of 5 nM DONs, previously stabilized at different N/P ratios, through a mobility shift assay after exposure to either no reductant (—) or 5% β-mercaptoethanol at 37°C for 1 hour. Ld: 1 kB ladder; Ctl: non-coated DON in FoB. Cy5 signal is shown in red, SybrSafe in blue.

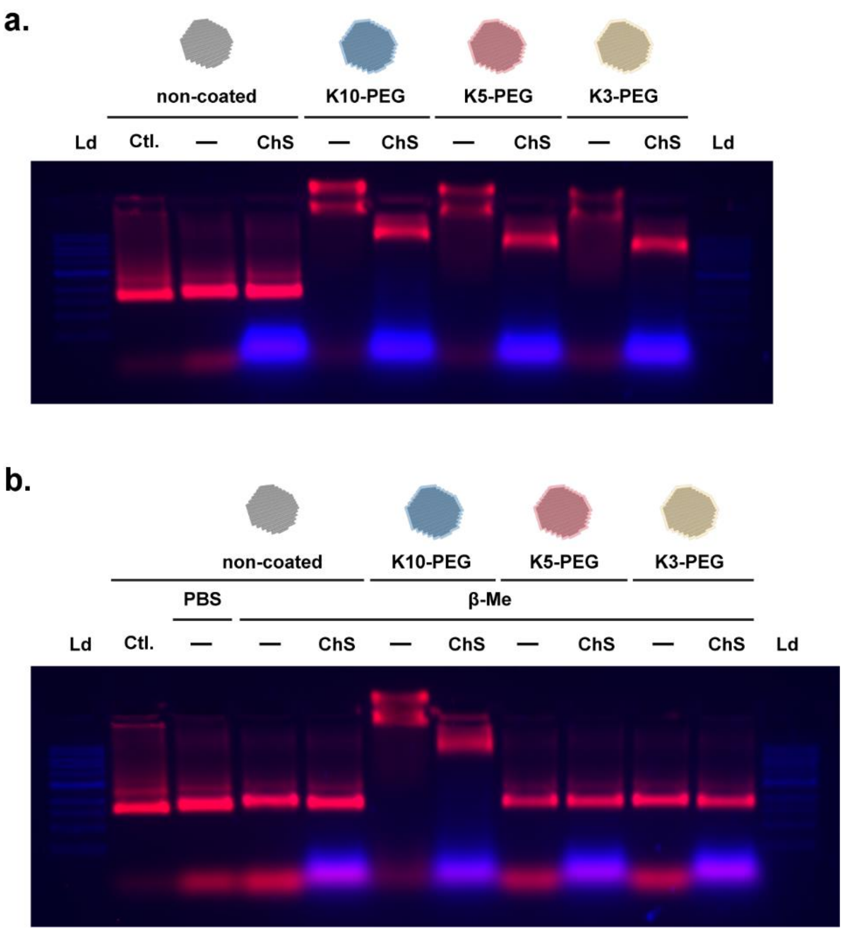


**Figure S9| Coating decomplexation AGE assay after exposure to chondroitin sulfate polyanion.**

1% agarose gel (90 min run) evaluating the decomplexation of 5 nM DONs, previously stabilized at different N/P ratios, through a mobility shift assay after the addition of 1 µL of chondroitin sulfate (0.3 M) for 1 hour. The top panel shows samples without prior exposure to reductive conditions, while the bottom panel shows samples after incubation with 5% β-mercaptoethanol at 37°C for 1 hour. Ld: 1 kB ladder; Ctl: non-coated DON in FoB. Cy5 signal is shown in red, SybrSafe in blue.
